# Resting-State Functional Connectivity Estimated With Hierarchical Bayesian Diffuse Optical Tomography

**DOI:** 10.3389/fnins.2020.00032

**Published:** 2020-01-31

**Authors:** Takatsugu Aihara, Takeaki Shimokawa, Takeshi Ogawa, Yuto Okada, Akihiro Ishikawa, Yoshihiro Inoue, Okito Yamashita

**Affiliations:** ^1^Graduate School of Human and Environmental Studies, Kyoto University, Kyoto, Japan; ^2^Neural Information Analysis Laboratories, Advanced Telecommunications Research Institute International, Kyoto, Japan; ^3^Cognitive Mechanisms Laboratories, Advanced Telecommunications Research Institute International, Kyoto, Japan; ^4^Graduate School of Information Science, Nara Institute of Science and Technology, Nara, Japan; ^5^Medical Systems Division, Research and Development Department, Shimadzu Corporation, Kyoto, Japan; ^6^RIKEN Center for Advanced Intelligence Project, Tokyo, Japan

**Keywords:** resting-state functional connectivity (RSFC), near-infrared spectroscopy (NIRS), diffuse optical tomography (DOT), hierarchical Bayesian estimation algorithm, minimum norm algorithm

## Abstract

Resting-state functional connectivity (RSFC) has been generally assessed with functional magnetic resonance imaging (fMRI) thanks to its high spatial resolution. However, fMRI has several disadvantages such as high cost and low portability. In addition, fMRI may not be appropriate for people with metal or electronic implants in their bodies, with claustrophobia and who are pregnant. Diffuse optical tomography (DOT), a method of neuroimaging using functional near-infrared spectroscopy (fNIRS) to reconstruct three-dimensional brain activity images, offers a non-invasive alternative, because fNIRS as well as fMRI measures changes in deoxygenated hemoglobin concentrations and, in addition, fNIRS is free of above disadvantages. We recently proposed a hierarchical Bayesian (HB) DOT algorithm and verified its performance in terms of task-related brain responses. In this study, we attempted to evaluate the HB DOT in terms of estimating RSFC. In 20 healthy males (21–38 years old), 10 min of resting-state data was acquired with 3T MRI scanner or high-density NIRS on different days. The NIRS channels consisted of 96 long (29-mm) source-detector (SD) channels and 56 short (13-mm) SD channels, which covered bilateral frontal and parietal areas. There were one and two resting-state runs in the fMRI and fNIRS experiments, respectively. The reconstruction performances of our algorithm and the two currently prevailing algorithms for DOT were evaluated using fMRI signals as a reference. Compared with the currently prevailing algorithms, our HB algorithm showed better performances in both the similarity to fMRI data and inter-run reproducibility, in terms of estimating the RSFC.

## Introduction

Brain consists of spatially distributed regions that have their own function, but these regions are functionally connected, that is, they continuously send information to each other. Recent progress in the acquisition and analysis of functional neuroimaging data has made it possible to explore functional connectivity in the human brain. Functional connectivity is defined as a temporal correlation of neuronal activation patterns between anatomically distant brain regions and has been assessed using various non-invasive functional neuroimaging modalities including functional magnetic resonance imaging (fMRI), magnetoencephalography (MEG) and electroencephalography (EEG). In particular, functional connectivity under resting conditions (resting-state functional connectivity, RSFC) has attracted widespread attention in neuroscience (Biswal et al., 1995; Greicius et al., 2003). One of the reasons for this might be that a growing body of studies has reported altered levels of functional connectivity in neurological and psychiatric brain disorders, including Alzheimer’s disease, depression, dementia and schizophrenia (van den Heuvel and Hulshoff Pol, 2010).

Most RSFC studies use fMRI because of its high spatial resolution. For example, the first direct evidence for the default mode network (DMN) was demonstrated using resting-state fMRI data based on seed-based correlation analysis (Greicius et al., 2003). The dorsal attention network (DAN), the resting-state network antagonistically coupled with the DMN, were also identified using resting-state fMRI data with the seed-based approach (Fox et al., 2005). There is a more sophisticated approach than the seed-based correlation analysis, the use of spatial independent component analysis (ICA). Spatial ICA is a widely used method for decomposing fMRI data into signal and noise components and was implemented using the Group ICA of fMRI Toolbox (GIFT)^1^. Multiple resting-state networks including DMN can be easily identified by applying spatial ICA to resting-state fMRI data (Jafri et al., 2008), and therefore the use of this approach is increasing. Despite growing use of fMRI in RSFC studies, fMRI has dis-advantages of high cost and low portability. In addition, fMRI may not be safe or appropriate for people (1) with metal or electronic implants in their bodies (such as pacemakers, cochlear implants, metallic tattoos, etc.) because MRI involves exposure to strong magnetic fields and induced electric fields, (2) with claustrophobia because subjects are required to enter narrow scanner tube and (3) who are or may be pregnant because the risk of exposure to magnetic fields for the fetus is still unknown.

Functional near-infrared spectroscopy (fNIRS) is a non-invasive optical imaging technique that measures changes in both oxygenated (oxy-) and deoxygenated (deoxy-) hemoglobin (Hb) concentrations based on changes in light absorption at multiple wavelengths, whereas fMRI mainly measures changes in deoxy-Hb concentrations, referred to as the blood-oxygen-level-dependent (BOLD) signals. Because fNIRS is free of above disadvantages in fMRI, it can be used as an alternative human brain mapping technique for situations in which fMRI is contraindicated. Rather than an alternative to fMRI, fNIRS would provide even additional information, because fNIRS creates images of both oxy- and deoxy-Hb simultaneously (as described above, the BOLD signal is mostly sensitive to deoxy-Hb) and has a higher sampling rate than fMRI does (>10 Hz with fNIRS, whereas ∼0.5 Hz with fMRI). Thanks to these advantages, fNIRS has been used to investigate RSFC. Lu et al. (2010), one of the earliest resting-state fNIRS studies, demonstrated that, using seed-based correlation analysis, RSFC maps over the sensorimotor and auditory cortexes were consistent with those of previous fMRI findings. Furthermore, Duan et al. (2012) and Sasai et al. (2012) examined relationship of RSFCs between fNIRS and fMRI by simultaneously recording these signals and demonstrated that fNIRS can be used to collect information regarding RSFC defined in fMRI. As for the approach to estimate RSFC, Zhang et al. (2010) compared a spatial ICA with the conventional seed-based correlation approach with respect to the estimation of RSFC from fNIRS data and demonstrated the superior performance of spatial ICA with higher sensitivity and specificity, especially in the case of higher noise level (Zhang et al., 2010). Despite the success these resting-state fNIRS studies achieved, current standard fNIRS imaging (i.e., optical topography that is two-dimensional image based on the spatial interpolation method) has several disadvantages. First, fNIRS imaging uses sparse arrangements of source and detector optodes (and therefore measurement channels are also sparsely arranged) and therefore the positions of the measurement channels do not always overlap the real activation foci. Therefore, the spatial resolution of fNIRS imaging is low compared to fMRI. Second, the positions of the measurement channels relative to brain anatomy vary among subjects, and also among runs within the same subjects when the runs were performed on different days, resulting in reduced reliability of comparison among subjects and runs. Third, fNIRS signals are, in most cases, degraded by the hemodynamic changes in the scalp layer. Changes in scalp hemodynamics sometimes exceed those in cortical hemodynamics (Takahashi et al., 2011). In these three problems, the third one was dealt with by several studies that succeeded to reduce scalp artifacts with the use of principal component analysis (PCA) (Zhang et al., 2005), independent component analysis (ICA) (Kohno et al., 2007), short-distance channel regression (Zeff et al., 2007; Eggebrecht et al., 2014) or combination of PCA and multi-distance probe arrangement (Sato et al., 2016). However, there is a way to solve all the three problems at once, a method called diffuse optical tomography (DOT).

Diffuse optical tomography is an advanced technique to reconstruct three-dimensional images showing changes in cerebral hemodynamics. The technique follows the strategy to use high-density DOT grids, which bring about overlapping measurements at multiple source-detector separation distances. The use of overlapping measurements improves spatial resolution in the head surface direction. In addition, different measurement distances provide information about different depths. This is because the penetration depth of the light increases with the source-detector separation distance. Depending on the source-detector distance and the subject’s scalp/skull thickness, the light may or may not sufficiently penetrate through the superficial layers (scalp) to the deeper layers (brain tissue) (Rupawala et al., 2018). There are two stages to obtain DOT images. The first stage is forward modeling where the measurement process is simulated using a head model and physical laws. The second stage is image reconstruction where the hemodynamic changes inside the head medium are estimated from fNIRS signals by inverting the forward model. The image reconstruction problem is formulated as a linear inverse problem (Boas et al., 2004), which is ill-posed and therefore requires *a priori* information to constrain possible solutions. One approach to solve the inverse problem is the regularization. In the DOT algorithm based on the regularization (Zeff et al., 2007; Eggebrecht et al., 2014), a DOT image is obtained by minimizing a cost function consisting of the data fitting term and constraint terms representing *a priori* information. Another is the Bayesian approach, which uses a probabilistic model of observations and constraints called the likelihood function and prior distribution, respectively (Guven et al., 2005; Shimokawa et al., 2012).

Although there were a variety of image reconstruction algorithms, no research had proposed a DOT algorithm to accurately reconstruct both the scalp and cortical activities simultaneously. Most studies took a two-process approach: (1) hemodynamic changes in the superficial layers, including scalp and skull, were removed from all measurements and (2) a DOT image reconstruction method was applied to the denoised data in order to estimate only the cortical activity. Most artifact-removal methods used in the first process were based on an assumption that the temporal patters in the hemodynamic changes of the superficial layers are homogeneous over the whole head (e.g., Kohno et al., 2007) or they are similar to those of short-distance channels (Eggebrecht et al., 2014). However, the artifact-removal methods based on the above assumption would not work well if the systemic interference occurring in the superficial layers of the human head is inhomogeneous across the surface of the scalp as reported in Gagnon et al. (2012, 2014) or if hemodynamic changes in the superficial layers are highly correlated with those in the cortex (though see Kirilina et al., 2012; Funane et al., 2014; for methods to overcome this). To avoid these weak points in the artifact-removal methods based on temporal information, we recently proposed a method that uses the spatial information of the optical paths of all observation channels, which are not affected by temporal inhomogeneity or correlation, in order to remove the scalp hemodynamics (Shimokawa et al., 2013). The method is an expanded version of the previously proposed hierarchical Bayesian (HB) DOT algorithm (Shimokawa et al., 2012) and is able to reconstruct both the scalp and cortical activities simultaneously.

In the first version of our Bayesian DOT algorithm (Shimokawa et al., 2012), we introduced sparse regularization to improve the depth accuracy and the spatial resolution and verified its performance with phantom experiments. Then, in the expanded version (Shimokawa et al., 2013), we introduced different types of regularization for the cortex and the scalp, sparse and smooth regularization to cortical and scalp’s hemodynamic changes, respectively, and validated the proposed method through both two-layer phantom experiments and MRI-based head-model simulations. Furthermore, we have conducted real human experiments with movement tasks and confirmed the performance of the HB DOT on imaging task-related functional responses (Yamashita et al., 2016). The present study therefore aims to investigate whether the HB DOT is used successfully to estimate spontaneous changes in cortical hemodynamics instead of task-related changes. We conducted real human experiments to record resting-state fNIRS signals from bilateral frontal and parietal areas using high-density probe array with multiple-distance channels, which required a major improvement of measurement (i.e., development of a custom-made holder that stably fits to the scalp). Then, we calculated the RSFC from the estimates of cortical hemodynamic changes with HB DOT. We also acquired resting-state fMRI data and used it as a reference in order to validate the performance of our algorithm. In addition, we compared the performance of our method with that of the two-process approach, which is the currently prevailing method for DOT. As for the DOT algorithms used in the two-process approach, we adopted the modified depth-compensation minimum-norm algorithm (abbreviated as MN) and the current standard method developed in Washington University (named in this study as MN-WU) (Eggebrecht et al., 2014), in line with our previous study on imaging task-related activities (Yamashita et al., 2016). Because there are still few studies on RSFC using DOT (White et al., 2009; Eggebrecht et al., 2014), the present study will provide important information to the relevant research areas.

## Materials and Methods

**FIGURE 1:**
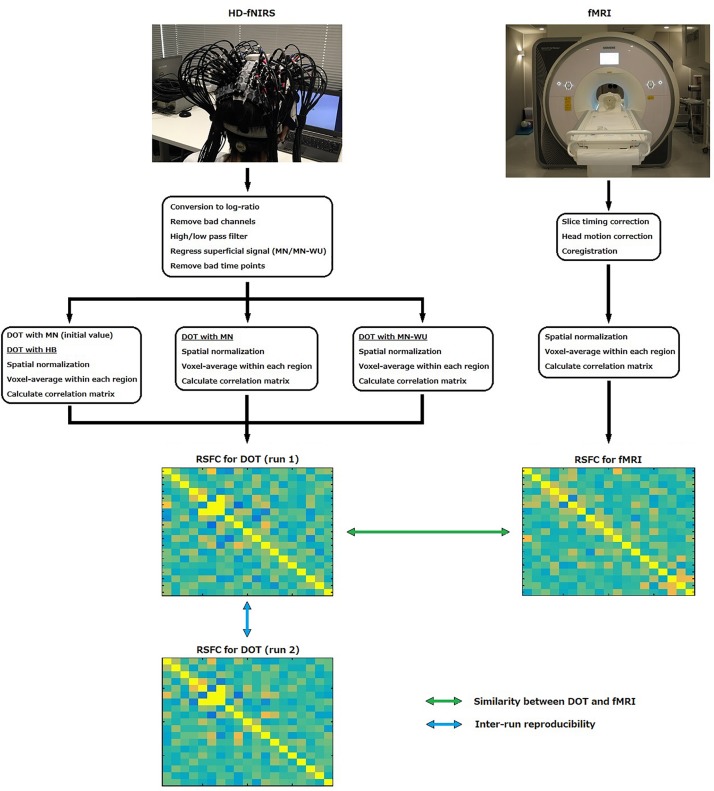
Schematic of the processing stream for fNIRS and fMRI data.

To obtain resting-state brain activity, fMRI and fNIRS data were recorded during resting state on different days. fMRI data was used as a reference. fNIRS signals were passed through DOT analyses to reconstruct three-dimensional images of changes in cerebral hemodynamics. Three different DOT algorithms, HB, MN and MN-WU were compared in terms of resting-state connectivity. Schematic of the processing stream for fNIRS and fMRI data are shown in Figure 1.

### Subjects

Twenty healthy male subjects aged between 21 and 38 participated in both fMRI and fNIRS experiments on different days. All of the subjects, except for one subject who is one of the authors, were paid for their participation. None reported history of neurological or psychiatric disorders. All subjects gave written informed consent to participate in the experimental procedures, which were approved by the ATR Review Board Ethics Committee.

### Tasks

In the fMRI experiment, each subject undergone a 10 min resting state condition in which he/she was instructed to stay still, to stay awake, to fixate on the crosshair, and not to think about specific things.

In the fNIRS experiment, each subject undergone a two-back working memory task (took about 15 min) and two 10 min resting state conditions similar to that in the fMRI experiment. The resting state conditions were undergone before and after the WM task condition. We did not use the WM data in the present study.

### MRI and fMRI Data Acquisition

Subjects lay down in an MRI scanner. Structural MR images were acquired for construction of individual head models, and functional images were acquired for evaluation of the reconstructed DOT images. All MRI data were recorded using a 3T MRI scanners, MAGNETOM Trio Tim, MAGNETOM Verio, MAGNETOM Prisma (Siemens Medical Systems, Erlangen, Germany). The acquisition parameters for T1-weighted images were as follows: repetition time (TR) = 2,300 ms, time of echo (TE) = 2.98 ms, flip angle = 9°, slice thickness = 1 mm, field of view (FOV) = 256 mm, imaging matrix = 256 × 256, inversion time (TI) = 900 ms. The acquisition parameters for echo-planar images (EPIs) were as follows: TR = 2,500 ms, TE = 30 ms, flip angle = 80°, slice thickness = 3.2 mm, FOV = 212 mm, imaging matrix = 64 × 64 mm.

### NIRS Data Acquisition

**FIGURE 2:**
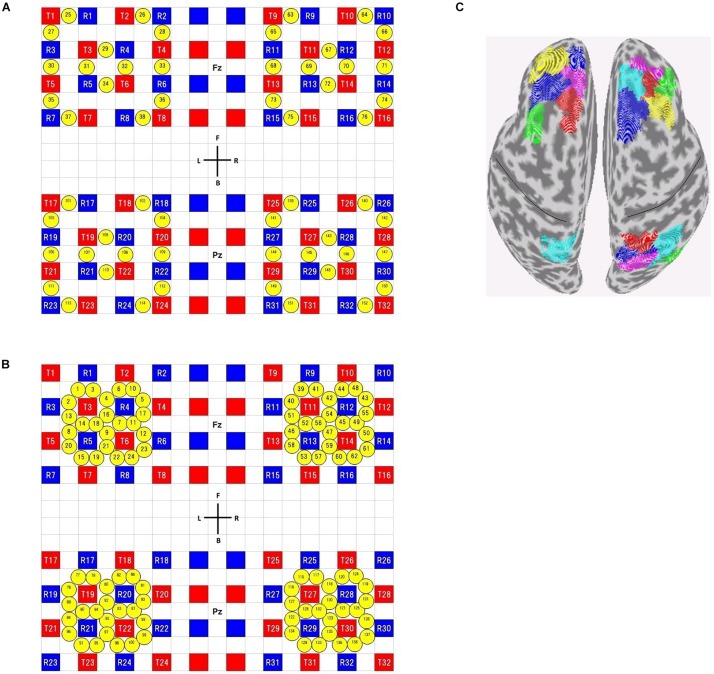
Channel configuration **(A,B)** and *available* ROIs on the brain surface **(C)**. **(A)** Configuration of the short-distance channels, **(B)** configuration of the long-distance channels. Red and blue squares represent source and detector positions, respectively. Yellow circles represent measurement channel positions. Note that, in long-distance channels, some channels (for example, ch1 and ch3) overlap each other in real situation, though not precisely described as such. A custom-made holder was divided into two parts. One is used to cover frontal areas and its center positioned on Fz (according to the international 10–20 system). The other is used to cover parietal areas and its center positioned on Pz. **(C)** *available* ROIs for Shen’s atlas (see section “**Calculation of Resting-State Connectivity**” for the definition of *available* ROIs). The black lines indicate the central sulci.

Subjects were seated in a comfortable reclining armchair. fNIRS data were acquired using commercial NIRS equipment (SMARTNIRS, Shimadzu Corp., Japan) with probes whose shapes were customized for high-density (HD) measurements. Using a custom-made holder, 32 source and 32 detector probes were placed on the scalp to cover bilateral frontal and parietal areas. We adopted four 4 × 4 square arrays (Figures 2A,B), where the first- and second-shortest distances between source and detector probes were 13 and 29 mm, respectively. We used all of the first- and second-nearest neighbor measurement pairs, which provided 56 ‘first’ and 96 ‘second’ channels, respectively, resulting in a total of 152 measurement channels.

Just after the fNIRS recording, the surface image of the subject’s face, the positions of the three fiducial markers (nasion, left and right preauricular points) and the probe positions were acquired with a hand-held laser scanner and a stylus marker (FastSCAN; Polhemus, United States), for the co-registration of the fNIRS data to the T1-MRI.

Three near-infrared beams (wavelength 780, 805, and 830 nm) were irradiated and detection beams sampled at 18.5 Hz were used to calculate Δ[oxy-Hb] and Δ[deoxy-Hb].

### fMRI Data Processing

fMRI signals were processed using SPM12 (the Wellcome Centre for Human Neuroimaging). The first four volumes were discarded to allow for T1 equilibration. The remaining data were corrected for slice timing and realigned to the mean image of that sequence to compensate for head motion. Next, the structural image was co-registered to the mean functional image and segmented into three tissue classes (gray matter, white matter, and cerebrospinal fluid) in the standard Montreal Neurological Institute (MNI) space. Using associated parameters, the functional images were spatially normalized into the MNI space and resampled in a 2 × 2 × 2 mm grid. Finally, they were spatially smoothed using an isotropic Gaussian kernel of 8 mm full-width at half maximum (FWHM).

### fNIRS Data Preprocessing

Prior to DOT analysis, the following preprocessing was applied to fNIRS signals.

(1) Convert voltage data into log-ratios using a base-10 logarithm.
(2) Calculate the coefficient of variation (CV, in%) of each channel for each wavelength, where CV = 100 × σ/μ, σ is the signal standard deviation, and μ is the mean signal level. Then, remove the bad channels which had CVs exceeding 15% (Piper et al., 2014) at least one of three wavelength or were saturated [the mean number of rejected channels was 13.0 ± 12.2 (SD)].
(3) Apply a high-pass filter (Butterworth filter of order 3, cutoff 0.009 Hz) and a low-pass filter (Butterworth filter of order 7, cutoff 0.08 Hz).
(4) Remove scalp hemodynamics from the filtered data, only in case of DOT image reconstruction with the MN and MN-WU algorithms. In this process, the global average of all the ‘first’ channels other than the bad channels is regarded as the scalp dynamics and regressed out. This process is omitted in case of DOT image construction with the HB algorithm.
(5) Remove the bad time points when absolute signal amplitudes exceeded more than three standard deviations from the mean at least one of the “first” channels [the mean number of removed time points was 590 ± 247 (SD) (roughly equal to 32 s)].

### DOT Forward Model Construction

The DOT forward model was constructed in the following way. First, an individual head model was constructed by segmenting their T1 structural image into five tissue layers [scalp, skull, cerebrospinal fluid (CSF), gray matter, and white matter], using FreeSurfer software^2^. Positions of the fNIRS probes were then co-registered to the head model using an affine transformation. The rotation and translation parameters of the affine transformation were optimized so that a subject’s facial surface measured by the laser scanner fitted that extracted from the T1 anatomical image. Next, the photon migration process inside the head was simulated with Monte Carlo simulation software MCX (Fang and Boas, 2009) with 10^9^ photons. We used tissue optical parameters common to all three wavelengths (Table 1), as presented in a previous study (Fang, 2010). Finally, the sensitivity matrix, which relates the absorption changes in the head tissue voxels to the light intensity changes at the source-detector pairs, was computed with Rytov approximation to the MCX results (Shimokawa et al., 2012). For computation of the sensitivity matrix, the 1 × 1 × 1 mm voxel space was down-sampled to 4 × 4 × 4 mm voxel space in the reconstructed images. The image reconstruction region included the scalp and cortical voxels inside a 28-mm-deep cuboid, whose surface was a square along the scalp surface. This was obtained by extending the diagonals of the 5 × 5 NIRS probe square by a factor of 1.5.

**TABLE 1:** Optical parameters in various head tissue types (common to all three wavelengths of 780, 805, and 830 nm).

**Tissue types**	**Absorpt. Coeff. μ_a_ (mm^–1^)**	**Scattering Coeff. μ_s_ (mm^–1^)**	**Anisotropy (g)**	**Refract. Index (n)**
Scalp and skull	0.019	7.8	0.89	1.37
CSF	0.004	0.009	0.89	1.37
Gray matter	0.02	9.0	0.89	1.37
White matter	0.08	40.9	0.84	1.37

### DOT Image Reconstruction With the HB Algorithm

DOT image reconstruction with the HB algorithm has two steps. In the first step, DOT image is reconstructed from the preprocessed fNIRS signals, using the modified version of the depth-compensation minimum norm image reconstruction algorithm (MN). In the next step, the DOT image is refined using the iterative algorithm with the MN DOT image obtained in the first step as an initial value and a prior. Note that the preprocessed fNIRS signals used in both steps are not passed through the scalp hemodynamics removal (the 4th process in section “fNIRS data preprocessing”).

For the HB DOT image reconstruction in the second step, we used the HB model presented in Yamashita et al. (2016). The hierarchical prior distribution has mean and confidence (or reliability) parameters. The mean parameter $\bar{\lambda_{0\,ı}}$ were the mean square values of the solutions obtained from the MN DOT in the first step. The confidence parameter γ_0_ controls the width of the hierarchical prior distribution (the variance of the hierarchical prior distribution is inversely proportional to the confidence parameter); large γ_0_ narrows the hierarchical prior distribution around the mean value $\bar{\lambda_{0\,ı}}$, and the estimation depends more critically on the solutions obtained from MN DOT in the first step. The confidence parameter γ_0_ was set to L × 0.1, where L is data length, on the basis of our experience. But, we also tried the following settings; γ_0_ = L × 0.01, L × 0.001, L × 0.0001. For the detail of the HB algorithm, see Yamashita et al. (2016).

### DOT Image Reconstruction With the MN/MN-WU Algorithms

The DOT image with the MN algorithm and that with the MN-WU algorithm were also computed for comparison. As mentioned in section “fNIRS data preprocessing,” in both MN and MN-WU algorithms, DOT image is reconstructed from the preprocessed fNIRS signals whose scalp hemodynamics were removed by regressing out the averaged ‘first’ channel data.

As for the MN-WU algorithm, the spatially variant parameter was set to β = 0.1 and the regularization parameter α was automatically determined by maximizing the marginal likelihood of each data set (see Culver et al., 2003).

As for the MN algorithms, the spatially variant parameter was set to β = *mean_νϵI20mm_*(ρ_2_)_*ν*_ where *I*_*20 mm*_ is a voxel index set whose depth from the scalp is around 20 mm. And the regularization parameter α was automatically determined by maximizing the marginal likelihood using all the measurements included in the I_task_. See the Appendix of Yamashita et al. (2016) for the mathematical details.

### Calculation of Resting-State Connectivity

Irrespective of the DOT algorithm, the reconstructed DOT image was passed through a spatial normalization into the standard MNI space and spatial smoothing with a Gaussian kernel of 8 mm full width at half maximum (FWHM), before calculation of resting-state functional connectivity (RSFC). These processes were done with SPM12.

The RSFC for both fMRI and DOT was obtained in the following way, using spatially normalized fMRI and DOT images, respectively. First, all cortical voxels were categorized into 278 regions of interest (ROI), based on functional-connectivity-based atlas (Shen et al., 2013). Then, voxels with sensitivity values of more than 0.5 for all subjects were regarded as *sensitive* voxels, and ROIs including more than or equal to 10 *sensitive* voxels were regarded as *available* ROIs (Figure 2C for Shen’s atlas). Third, for each *available* ROI, timeseries of all *sensitive* voxels within the ROI were averaged. These mean timeseries were assumed to represent temporal activity of the corresponding ROI. Finally, partial correlations between all pairs of *available* ROIs were computed to make a correlation matrix. Partial correlation was used to reduce the influence of extra-neural components such as physiological noise signals due to spontaneous low-frequency oscillations, respiration and cardiac pulsation (Sakakibara et al., 2016). Note that all the following analyses were done for *sensitive* voxels and *available* ROIs.

The reason why we used Shen’s atlas rather than the widely used Brodmann-based automatic anatomic labeling (AAL) atlas is as follows. The AAL atlas uses Brodmann areas which are based on cytoarchitecture. This atlas is not ideal because of its coarse-grained nature (116 regions for the AAL atlas whereas 278 regions for the Shen’s atlas in our data) and the risk of including different functional areas within a single region, with the consequence that the resultant mean timeseries may not accurately represent any of the contributing timeseries. Shen’s atlas is developed to avoid this pitfall and will provide meaningful nodes (Shen et al., 2013), and therefore is suitable for our case.

For reference, we will present the corresponding results for the AAL atlas in the Supplementary Material.

### Comparison of Connectivity Matrices Among DOT Algorithms

In order to establish the superiority of the HB DOT in the estimation of RSFC, we compared correlation matrices among the DOT algorithms (HB, MN, MN-WU) in the following three ways.

Because it seems reasonable to use fMRI data as a reference, we first compared *similarity* of RSFCs between fMRI and DOT among three DOT algorithms, where correlation coefficient was used as a similarity measure. The comparison was done in the following way: (1) the lower triangular portion of correlation matrix, *C*_*low*_, was transformed to *z(C_*low*_)* by using Fisher’s z-transformation, (2) correlation coefficient of *z(C_*low*_)* between fMRI and DOT, *R*_*fMRI–DOT*_, was calculated, (3) the correlation coefficient *R*_*fMRI–DOT*_ was transformed to *z(R_*fMRI–DOT*_)* by using Fisher’s *z*-transformation, (4) differences in mean *z(R_*fMRI–DOT*_)* among three DOT algorithms were tested using one-way analysis of variance (ANOVA) followed by multiple comparisons with the Tukey–Kramer correction.

Second, we compared inter-run *reproducibility* of RSFC estimation among three DOT algorithms. The comparison was done in the following way: (1) the lower triangular portion of correlation matrix, *C*_*low*_, was transformed to *z(C_*low*_)* by using Fisher’s *z*-transformation, (2) correlation coefficient of *z(C_*low*_)* between run1 and run2, *R_*ses*__1__–ses__2_*, was calculated, (3) the correlation coefficient *R_*ses*__1__–ses__2_* was transformed to *z(R_*ses*__1__–ses__2_)* by using Fisher’s *z*-transformation, (4) differences in mean *z(R_*ses*__1__–ses__2_)* among three DOT algorithms were tested using one-way analysis of variance (ANOVA) followed by multiple comparisons.

As a complementary metric to Pearson’s correlation, intra-class correlation (ICC; McGraw and Wong, 1996) was also used for assessment of reproducibility across runs. Both single and average measures, i.e., ICC(C,1) and ICC(C,k), were calculated using MATLAB function *ICC* by Arash Salarian (available at MATLAB Central File Exchange).

**FIGURE 3:**
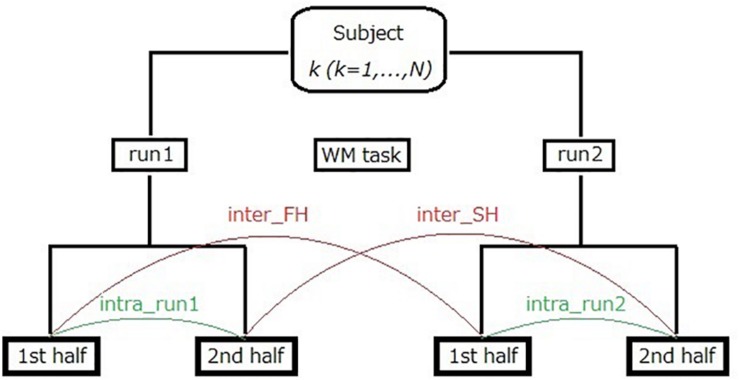
Illustration of intra-run reproducibility and half-length inter-run reproducibility calculations for fNIRS data. The number of subjects, N, was 20.

Third, we performed additional analysis on intra-run test–retest reliability assessment. In this analysis, resting-state fNIRS data recorded in each 10 min run was firstly divided into two segments of equal length [i.e., first half (FH) and second half (SH)]. Note that duration of each half is 5 min at most (the mean is about 4 min and 44 s) because bad time points were removed in the fNIRS data preprocessing (see section “fNIRS Data Preprocessing”). As shown in Figure 3, intra-run reproducibility was assessed using two pairs of datasets; run1-FH and run1-SH (intra_run1), as well as run2-FH and run2-SH (intra_run2). In addition, inter-run reproducibility (denoted by half-length inter-run reproducibility to distinguish it from the previous one using full-length data) was assessed with two pairs of datasets; run1-FH and run2-FH (inter_FH), as well as run1-SH and run2-SH (inter_SH). As measures for these reproducibilities, we used Pearson’s correlation, ICC(C,1) and ICC(C,k) again.

We compared intra-run reliability among functional images (i.e., fMRI vs. HB vs. MN vs. MN-WU). More specifically, differences in mean measures for intra-run reliability were tested using one-way ANOVA followed by multiple comparisons.

In addition, we compared intra-run reproducibility and half-length inter-run reproducibility. Because resting-state fNIRS was acquired before and after a WM task, this comparison will serve to investigate whether the task affected the resting-state connectivity.

## Results

### Similarity Between fMRI and DOT

**FIGURE 4:**
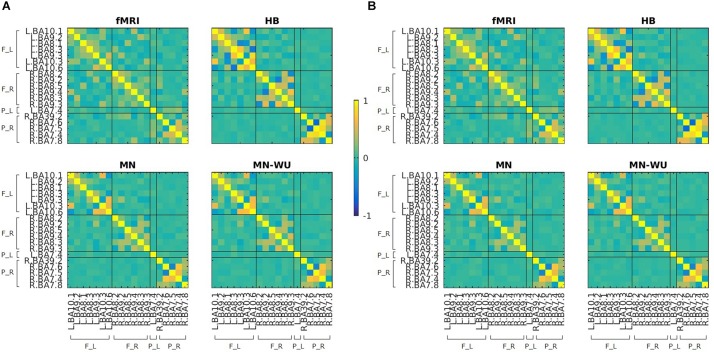
Mean maps for RSFCs of fMRI and DOT with HB, MN and MN-WU algorithms. Correlation matrices were averaged across subjects and runs, for **(A)** oxy-Hb and **(B)** deoxy-Hb in the case of DOT. F_L, left frontal area; F_R, right frontal area; P_L, left parietal area; P_R, right parietal area.

**FIGURE 5:**
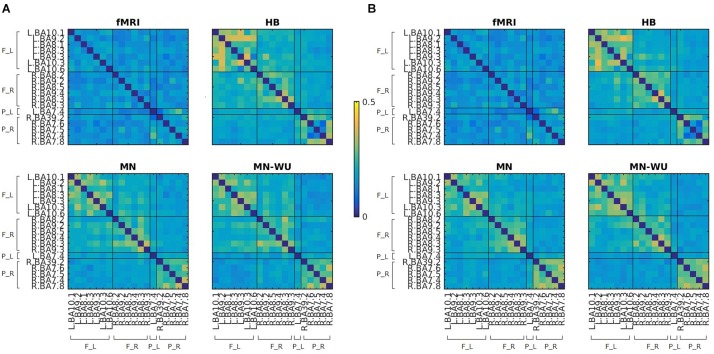
SD maps for RSFCs of fMRI and DOT with HB, MN and MN-WU algorithms. SDs of correlation matrices were computed across subjects and runs, for **(A)** oxy-Hb and **(B)** deoxy-Hb in the case of DOT. F_L, left frontal area; F_R, right frontal area; P_L, left parietal area; P_R, right parietal area.

Mean and SD maps for RSFCs of fMRI and DOT with HB, MN and MN-WU algorithms are shown in Figures 4, 5, respectively. The mean map showed a common tendency between fMRI and fNIRS (i.e., HB, MN, and MN-WU) that most pairs between adjacent ROIs had positive correlations, though some of them (e.g., R.BA7.5-R.BA7.6) had negative correlations. Additional findings from the mean map is that some of frontal-parietal pairs (e.g., R.BA7.8-R.BA9.4) and contralateral counterpart pairs (e.g., R.BA9.4-L.BA10.1) had positive correlations in fMRI, whereas such a tendency was weak for the DOT cases. As for the SD maps, variability across subjects was little in fMRI, whereas it was relatively large especially for the pairs between adjacent ROIs in fNIRS regardless of the DOT algorithms.

Similarity of RSFCs between fMRI and DOT for each run and DOT algorithm is summarized in Table 2. One-way ANOVA applied to data combined between runs revealed a significant difference among three DOT algorithms for both oxy- and deoxy-Hb [*F*_(__2_,_117__)_ = 4.27, *p* = 0.0162 for oxy-Hb; *F*_(__2_,_117__)_ = 7.69, *p* = 0.0007 for deoxy-Hb]. The *post hoc* Tukey’s HSD test revealed, for both oxy- and deoxy-Hb, that HB had significantly higher correlation values than both MN and MN-WU did (*p* < 0.05), but the difference of correlation values between MN and MN-WU was not significant.

**TABLE 2:** Similarity of correlation matrices between fMRI and DOT.

		**HB**	**MN**	**MN-WU**
oxy-Hb	run 1	0.34 ± 0.09	0.29 ± 0.10	0.29 ± 0.10
	run 2	0.35 ± 0.08	0.30 ± 0.08	0.30 ± 0.09
deoxy-Hb	run 1	0.33 ± 0.08	0.25 ± 0.11	0.26 ± 0.12
	run 2	0.34 ± 0.08	0.27 ± 0.11	0.25 ± 0.10

*Values of correlation coefficients are presented as mean ± standard deviation (SD).*

### Inter-Run Reproducibility, Intra-Run Reproducibility

**FIGURE 6:**
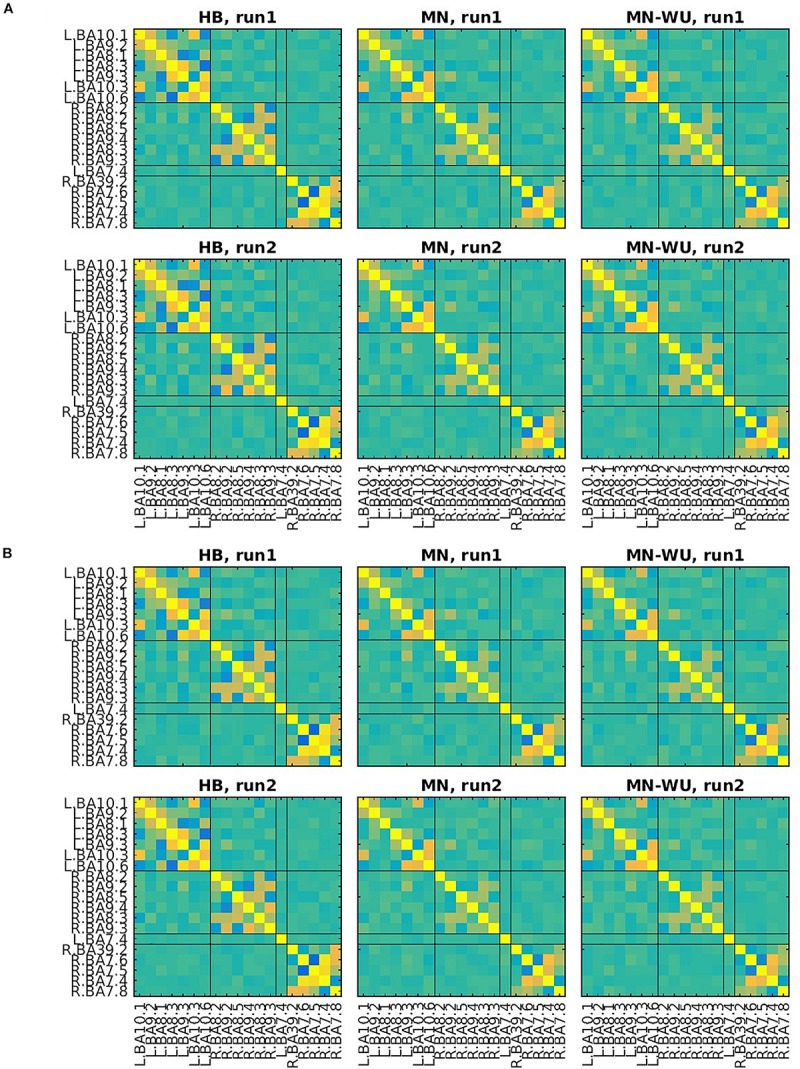
Mean maps for RSFCs are compared between run 1 and run 2. Correlation matrices are averaged across subjects for **(A)** oxy-Hb and **(B)** deoxy-Hb. The upper and lower rows correspond to run 1 and run 2, respectively. ROIs are displayed in the same order as those in Figures 4, 5.

**FIGURE 7:**
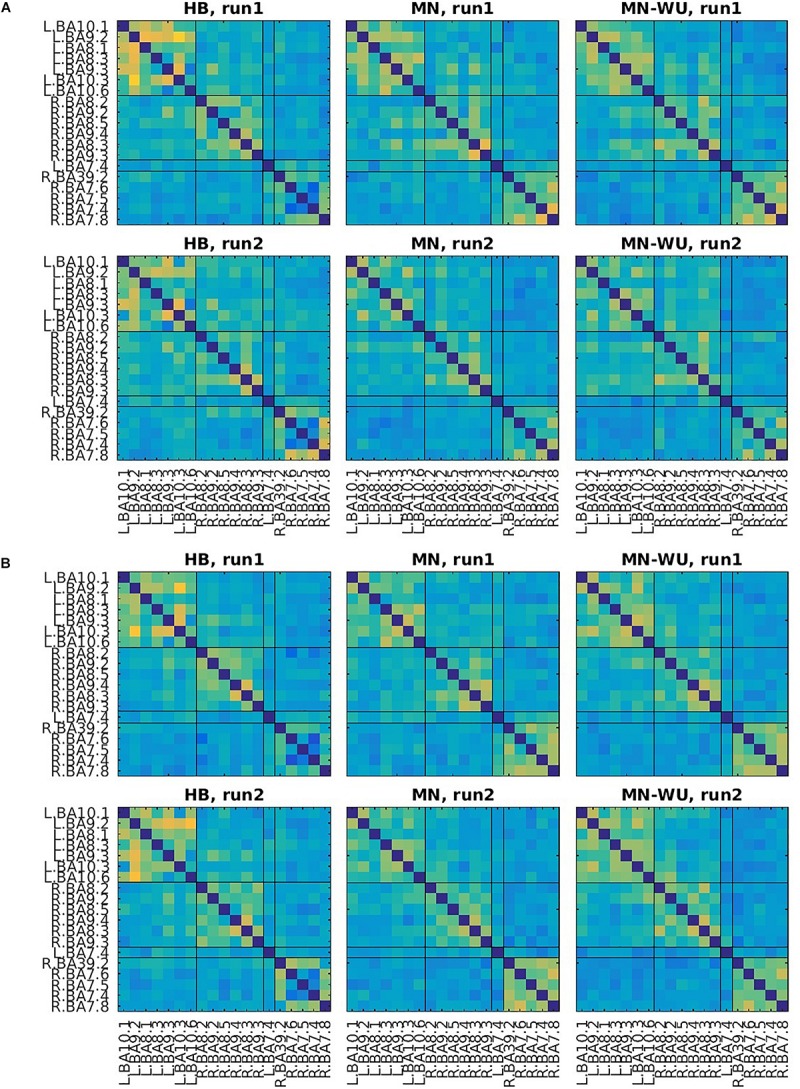
SD maps of RSFCs for run 1 and run 2. SDs of correlation matrices are computed across subjects for **(A)** oxy-Hb and **(B)** deoxy-Hb. The upper and lower rows correspond to run 1 and run 2, respectively. ROIs are displayed in the same order as those in Figure 6.

Mean and SD maps for RSFCs are compared between runs in Figures 6, 7, respectively. Mean ± SD of inter-run reproducibility (i.e., Pearson’s correlation and inter-class correlation of correlation matrices between run 1 and 2) is summarized in Table 3.

**TABLE 3:** Inter-run reproducibility.

		**HB**	**MN**	**MN-WU**
oxy-Hb	r	0.76 ± 0.06	0.61 ± 0.13	0.66 ± 0.12
	ICC(C,1)	0.76 ± 0.06	0.61 ± 0.13	0.65 ± 0.12
	ICC(C,k)	0.86 ± 0.04	0.75 ± 0.10	0.78 ± 0.09
deoxy-Hb	r	0.76 ± 0.07	0.60 ± 0.13	0.63 ± 0.15
	ICC(C,1)	0.75 ± 0.07	0.60 ± 0.13	0.62 ± 0.15
	ICC(C,k)	0.86 ± 0.04	0.74 ± 0.10	0.76 ± 0.12

*Values of Pearson’s correlation (r), ICC(C,1) and ICC(C,k) are presented as mean ± standard deviation (SD).*

As for Pearson’s correlation, one-way ANOVA revealed a significant difference among three DOT algorithms for both oxy- and deoxy-Hb [*F*_(__2_,_57__)_ = 11.0, *p* = 9.06 × 10^–5^ for oxy-Hb; *F*_(__2_,_57__)_ = 10.9, *p* = 9.89 × 10^–5^ for deoxy-Hb]. The *post hoc* Tukey’s HSD test revealed, for both oxy- and deoxy-Hb, that HB had significantly higher correlation values than both MN and MN-WU did (*p* < 0.05), but the difference of correlation values between MN and MN-WU was not significant.

As for the ICC metrices, the following results were obtained. First, for ICC(C,1), one-way ANOVA revealed a significant difference among three DOT algorithms for both oxy- and deoxy-Hb [*F*_(__2_,_57__)_ = 11.0, *p* = 9.30 × 10^–5^ for oxy-Hb; *F*_(__2_,_57__)_ = 9.81, *p* = 0.0002 for deoxy-Hb]. The *post hoc* Tukey’s HSD test revealed, for both oxy- and deoxy-Hb, that HB had significantly higher ICC(C,1) values than both MN and MN-WU did (*p* < 0.05), but the difference of ICC(C,1) values between MN and MN-WU was not significant.

Then, for ICC(C,k), one-way ANOVA revealed a significant difference among three DOT algorithms for both oxy- and deoxy-Hb [*F*_(__2_,_57__)_ = 10.5, *p* = 0.0001 for oxy-Hb; *F*_(__2_,_57__)_ = 8.61, *p* = 0.0005 for deoxy-Hb]. The *post hoc* Tukey’s HSD test revealed, for both oxy- and deoxy-Hb, that HB had significantly higher ICC(C,k) values than both MN and MN-WU did (*p* < 0.05), but the difference of ICC(C,k) values between MN and MN-WU was not significant.

Intra-run reproducibility (i.e., similarity of RSFCs between FH and SH) is summarized in Table 4.

**TABLE 4:** Intra-run reproducibility.

		**fMRI**	**HB**	**MN**	**MN-WU**
oxy-Hb	r	0.61 ± 0.08	0.57 ± 0.11	0.38 ± 0.16	0.41 ± 0.13
	ICC(C,1)	0.61 ± 0.08	0.56 ± 0.11	0.37 ± 0.15	0.40 ± 0.13
	ICC(C,k)	0.75 ± 0.06	0.71 ± 0.09	0.53 ± 0.16	0.56 ± 0.14
deoxy-Hb	r	0.61 ± 0.08	0.53 ± 0.10	0.38 ± 0.15	0.38 ± 0.15
	ICC(C,1)	0.61 ± 0.08	0.53 ± 0.10	0.37 ± 0.15	0.37 ± 0.15
	ICC(C,k)	0.75 ± 0.06	0.68 ± 0.09	0.52 ± 0.16	0.53 ± 0.15

*Values of correlation coefficients (*r*), ICC(C,1) and ICC(C,k) are presented as mean ± standard deviation (across subjects for fMRI; across subjects and runs for DOT).*

One-way ANOVA revealed that Pearson’s correlation value is significantly different among functional images [*F*_(__3_,_136__)_ = 27.26, *p* = 7.25 × 10^–14^ for oxy-Hb; *F*_(__3_,_136__)_ = 23.84, *p* = 1.84 × 10^–12^ for deoxy-Hb]. The *post hoc* Tukey’s HSD test revealed, for both oxy- and deoxy-Hb, that HB had significantly higher Pearson’s correlation than both MN and MN-WU did (*p* < 0.05), but the difference between HB and fMRI was not significant.

Similarly, according to one-way ANOVA, ICC(C,1) is significantly different among functional images [*F*_(__3_,_136__)_ = 27.41, *p* = 6.28 × 10^–14^ for oxy-Hb; *F*_(__3_,_136__)_ = 24.59, *p* = 8.94 × 10^–13^ for deoxy-Hb]. According to the Tukey’s HSD test, for both oxy- and deoxy-Hb, HB had significantly higher ICC(C,1) than both MN and MN-WU did (*p* < 0.05), but HB was not significantly different from fMRI. In addition, one-way ANOVA and *post hoc* analysis revealed that ICC(C,k) had similar tendency [one-way ANOVA, *F*_(__3_,_136__)_ = 25.00, *p* = 6.04 × 10^–13^ for oxy-Hb; *F*_(__3_,_136__)_ = 23.15, *p* = 3.61 × 10^–12^ for deoxy-Hb].

For the HB case, intra-run reproducibility (i.e., similarity of RSFCs between FH and SH) was compared with half-length inter-run reproducibility (i.e., similarity of RSFCs between run 1 and run 2 for the corresponding half) in Table 5. Two-sample *t*-test revealed that intra-run reproducibility was not significantly different from half-length inter-run reproducibility for Pearson’s correlation (*p* = 0.31 for oxy-Hb; *p* = 0.42 for deoxy-Hb), ICC(C,1) (*p* = 0.36 for oxy-Hb; *p* = 0.47 for deoxy-Hb), and ICC(C,k) (*p* = 0.33 for oxy-Hb; *p* = 0.48 for deoxy-Hb). Similar tendency was observed for both MN and MN-WU cases (detailed data not shown, but *p* > 0.30 in any case).

**TABLE 5:** Comparison of reproducibility between intra-run and half-length inter-run for HB.

		**Intra-run**	**Half-length inter-run**
oxy-Hb	r	0.57 ± 0.11	0.55 ± 0.12
	ICC(C,1)	0.56 ± 0.11	0.54 ± 0.12
	ICC(C,k)	0.71 ± 0.09	0.69 ± 0.10
deoxy-Hb	r	0.53 ± 0.10	0.51 ± 0.10
	ICC(C,1)	0.53 ± 0.10	0.51 ± 0.10
	ICC(C,k)	0.68 ± 0.09	0.67 ± 0.09

*Values are presented as mean ± standard deviation.*

### Effect of the Confidence Parameter γ_0_ on Connectivity

As mentioned in the Materials and methods, in the HB algorithm, the confidence parameter, γ_0_, represents the width of the hierarchical prior distribution, controlling how strong the HB method is affected by the prior information (the depth-weighted minimum norm estimation). In this subsection, the effect of the γ_0_ value on the connectivity estimation was examined for only Pearson’s correlation.

Similarity of RSFCs between fMRI and DOT for each run and γ_0_ value is summarized in Table 6. One-way ANOVA applied to data combined between runs revealed no significant difference among five γ_0_ values for both oxy- and deoxy-Hb [*F*_(__4_,_195__)_ = 1.26, *p* = 0.287 for oxy-Hb; *F*_(__4_,_195__)_ = 1.40, *p* = 0.237 for deoxy-Hb].

**TABLE 6:** Effect of γ_0_ value on similarity of correlation matrices between fMRI and DOT.

		**L × 10^0^**	**L × 10^–1^**	**L × 10^–2^**	**L × 10^–3^**	**L × 10^–4^**
oxy-Hb	run 1	0.34 ± 0.09	0.34 ± 0.09	0.33 ± 0.09	0.32 ± 0.10	0.30 ± 0.11
	run 2	0.35 ± 0.09	0.35 ± 0.08	0.35 ± 0.08	0.34 ± 0.09	0.32 ± 0.09
deoxy-Hb	run 1	0.33 ± 0.08	0.33 ± 0.08	0.32 ± 0.08	0.31 ± 0.09	0.29 ± 0.10
	run 2	0.35 ± 0.08	0.34 ± 0.08	0.34 ± 0.08	0.33 ± 0.08	0.31 ± 0.08

*Values of correlation coefficients are presented as mean ± standard deviation (SD). Each column corresponds to γ_0_ = L × 10^–*N*^, where L is the data length (L = 7774–8723, mean 8285). γ_0_ = L × 10^–1^ is the default setting.*

Mean ± SD of inter-run reproducibility (i.e., correlation values of correlation matrices between run 1 and 2) for each γ_0_ value is summarized in Table 7. One-way ANOVA revealed no significant difference among five γ_0_ values for both oxy- and deoxy-Hb [*F*_(__4_,_95__)_ = 0.58, *p* = 0.679 for oxy-Hb; *F*_(__4_,_95__)_ = 0.49, *p* = 0.744 for deoxy-Hb].

**TABLE 7:** Effect of γ_0_ value on inter-run reproducibility.

	**L × 10^0^**	**L × 10^–1^**	**L × 10^–2^**	**L × 10^–3^**	**L × 10^–4^**
oxy-Hb	0.76 ± 0.06	0.76 ± 0.06	0.76 ± 0.06	0.75 ± 0.07	0.73 ± 0.09
deoxy-Hb	0.75 ± 0.07	0.76 ± 0.07	0.75 ± 0.08	0.74 ± 0.09	0.72 ± 0.10

*Values of correlation coefficients are presented as mean ± standard deviation (SD). Each column corresponds to γ_0_ = L × 10^–*N*^, where L is the data length. γ_0_ = L × 10^–1^ is the default setting.*

## Discussion

The aim of this study was to evaluate our proposed HB DOT algorithm in terms of its performance to estimate the resting-state functional connectivity among brain regions, not task-related brain responses. We used fMRI data as a reference. In addition, we compared our method with other DOT algorithms (the MN and MN-WU), which adopt the two-process approach. Similarity (i.e., correlation coefficient) of the RSFCs between fMRI and DOT showed higher for the HB than both the MN and MN-WU, suggesting that DOT with the HB algorithm is more appropriate to a substitute for fMRI than those with the MN and MN-WU in estimating the resting-state functional connectivity as well as the task-related cortical responses (Yamashita et al., 2016). In addition, inter-run reproducibility (i.e., Pearson’s correlation and intra-class correlation coefficients of RSFCs between runs) showed higher for the HB than both the MN and MN-WU, suggesting that DOT with the HB algorithm is more reliable. In addition, mean values of both single- and average-measure ICC are far higher than 0.4, a criterion of sufficient reliability (Zhang et al., 2011), suggesting that DOT with the HB algorithm is highly reliable and comparable to fMRI. These results were true for not only deoxy-Hb but also oxy-Hb, which cannot be measured by fMRI.

We conducted additional analyses relating to intra-run reproducibility (i.e., similarity of RSFCs between FH and SH) for fNIRS data. As for the intra-run test-retest reliability, the HB had significantly higher intra-run reproducibility than both the MN and MN-WU for any intra-run reproducibility measure. In particular, both single- and average-measure ICC values for the HB were far higher than 0.4 and comparable to those for the fMRI. Thus, high reliability of DOT image with the HB algorithm was demonstrated in intra-run as well as inter-run analyses. Meanwhile, comparison of reproducibility between intra-run and half-length inter-run showed no significant difference. This result suggests that the WM task, conducted between the two resting-state fNIRS runs, did not affect RSFCs. According to Birn et al. (2013), the reliability of the resting-state fMRI connectivity estimates was low for the scan length less than 5 min. However, as described in section “Comparison of Connectivity Matrices Among DOT Algorithms,” mean scan lengths of both first and second halves of resting-state fNIRS recording were slightly less than 5 min in the present study. Thus, re-examination of results on intra-run analyses is desirable using data with sufficiently long scan lengths.

According to the SD maps for the RSFCs (i.e., Figure 5), variability across subjects was little in fMRI, whereas it was large in fNIRS regardless of the DOT algorithms. This difference suggests that the variability of the estimated DOT images across subjects is larger than that of the fMRI images. Such a large variability in DOT images may be due to errors in forward modeling such as probe coregistration error and head model error, variability in measurement condition (e.g., individual difference in a signal-to-noise ratio) and individual difference in optical parameters which cannot be precisely dealt in forward modeling. In addition, oxy-Hb or deoxy-Hb may not solely correspond to BOLD signals. Further work is required to explore the cause of this difference.

In the calculation of RSFCs, we used (1) Shen’s atlas rather than the widely used AAL atlas and (2) timeseries averaged across all sensitive voxels within each region. This is because we considered that each region of the AAL includes different functional areas because of its coarse-grained nature [116 regions for a whole brain, but 9 *available* regions in our case (Supplementary Figure S1)], whereas that of the Shen’s atlas includes a single functional area due to its fine-grained nature [order of 300 regions in a whole brain, but 19 *available* regions in our case (Figure 2C)] and therefore mean timeseries represent temporal activity of the ROI. In fact, as described in Supplementary Material (Section 3 Kendall’s *W* analysis) the chi-squared test suggested that time courses of all voxels in each region of the Shen’s atlas were concordant, supporting that the Shen’s atlas provides functional subunits and therefore mean timeseries represent temporal activity of the ROI. In addition, the Kendall’s *W* for the Shen’s atlas is significantly larger than that for the AAL atlas, supporting the validity of using the Shen’s atlas in the calculation of functional connectivity. These results are true, regardless of the DOT algorithms. Furthermore, the additional analysis with PCA (Section 4 Principal component analysis in Supplementary Material) also confirmed the validity of using both voxel-averaged timeseries and Shen’s atlas.

As described in the previous paragraph, the AAL atlas is not ideal. However, the chi-squared test also failed to reject the null hypothesis that there is no concordance among all voxels in the region of the AAL atlas (*p* < *0.05* for all *available* ROIs). We therefore calculated the AAL counterpart on both (1) similarity between fMRI and DOT and (2) inter-run reproducibility, for your reference (see Supplementary Material for details). The similarity measure revealed no significant difference among three DOT algorithms (HB vs. MN vs. MN-WU), which is inconsistent with the Shen’s case. On the contrary, the inter-run reproducibility for the HB tended to be higher than that for the MN and MN-WU, which is consistent with the Shen’s case. Thus, the AAL counterpart was not always consistent with the Shen’s results. Although we do not have evidence that each region of the AAL atlas includes different functional areas, it is obvious that the Shen’s atlas is more suitable. Thus, the results for the Shen’s atlas case seem to be more reliable.

The confidence parameter γ_0_ in the HB DOT determines the balance between the data and the prior information. As far as we investigated in the present study, γ_0_ did not significantly affected either similarity of RSFCs between fMRI and DOT or inter-run reproducibility of RSFCs. However, both correlation values slightly decreased by setting γ_0_ the lowest value (L × 10^–4^). This may be consistent with the previous finding that a very low γ_0_ results in incorrectly localized sparse DOT images in some subjects in real experimental data which has a low signal-to-noise ratio (Yamashita et al., 2016). At present, we do not have a technique to determine the optimal γ_0_ value. Further work is required to find it. In the present study, we did not adopt the prevailing approach in the resting-state fMRI studies, spatial ICA. One of the reasons for this is that spatial ICA is suited for whole brain analysis whereas fNIRS data in the present study covered only frontal and parietal areas. Applying spatial ICA to whole brain DOT images will be a challenging future work.

## Conclusion

The present study showed that our HB algorithm can be used as an alternative to fMRI, in estimating resting-state functional connectivity as well as task-related responses. We also demonstrated its superiority over the current standard DOT algorithms. Although fNIRS data in the present study covered only frontal and parietal areas due to the experimental limitation of the high-density measurement, it is desired to cover wider areas of the cortex (ideally whole brain). Recently, we showed that multi-directional measurement has the ability to accomplish DOT without requiring high-density measurement (Shimokawa et al., 2016). It would be interesting to estimate RSFC for more widespread cortical areas using multi-directional DOT in future work.

## Data Availability Statement

The raw data supporting the conclusions of this article will be made available by the authors, without undue reservation, to any qualified researcher.

## Ethics Statement

The studies involving human participants were reviewed and approved by the ATR Review Board Ethics Committee. The patients/participants provided their written informed consent to participate in this study.

## Author Contributions

TA, TS, TO, YO, AI, YI, and OY designed the study. TA, TS, TO, YO, and OY collected the data. TA analyzed the data. TA, TS, and OY wrote and reviewed the manuscript.

## Conflict of Interest

Although the authors AI and YI were employed by the company Shimadzu Corporation, they were not involved in the comparison of several DOT algorithms and therefore the results in the present study cannot be biased.

The remaining authors declare that the research was conducted in the absence of any commercial or financial relationships that could be construed as a potential conflict of interest.

## Footnotes

1 http://trendscenter.org/software/gift/

2 https://surfer.nmr.mgh.harvard.edu
