# Supplementary material for: Resting-State Functional Connectivity Estimated With Hierarchical Bayesian Diffuse Optical Tomography
Source: Front Neurosci. 2020 Jan 31;14:32. doi: 10.3389/fnins.2020.00032 (PMC7005139; doi:10.3389/fnins.2020.00032)
Supplement: Supplementary file 1 [file Data_Sheet_1.docx]

# Supplementary Material

In the Supplementary Material (**Sec 1 The AAL counterpart; similarity between fMRI and DOT**, **Sec 2 The AAL counterpart; inter-run reproducibility**), results on some of the main analyses applied to the data for the AAL atlas case were shown. In addition, we confirmed the validity of using both voxel-averaged timeseries and Shen’s atlas in **Sec 3 Kendall’s W analysis** and **Sec 4 Principal component analysis** of the Supplementary Materials.

Figure S1 shows the *available* ROIs for the AAL atlas. In the case of our data, there are only 9 *available* ROIs for the case of the AAL atlas, whereas 19 *available* ROIs for the Shen’s case.


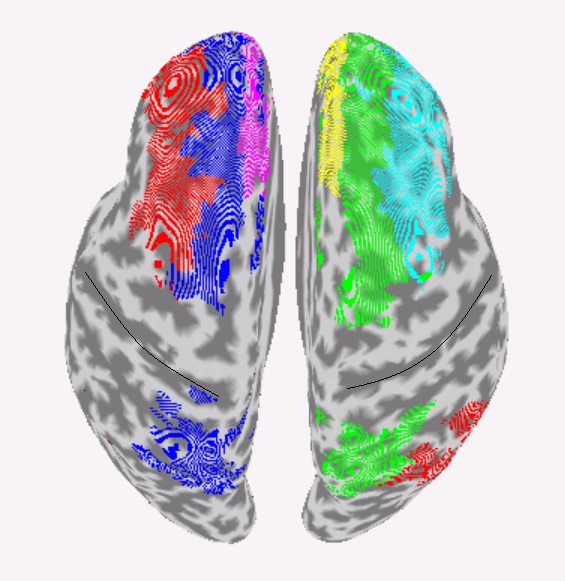


Figure S1. The *available* ROIs on the brain surface for the AAL atlas. The black lines indicate the central sulci.

**1 The AAL counterpart; similarity between fMRI and DOT**

As described in the main text, AAL atlas is not ideal, but, for your reference, we show the corresponding results below.

Mean and SD maps for RSFCs of fMRI and DOT with HB, MN and MN-WU algorithms are shown in Fig. S2 and S3, respectively and similarity (Pearson’s correlation) of RSFCs between fMRI and DOT for each run and DOT algorithm is summarized in Table S1. One-way ANOVA applied to data combined between runs revealed that there is no significant difference among three DOT algorithms (F_(2,117)_ = 1.71, p > 0.05 (p = 0.186) for oxy-Hb; F_(2,117)_ = 1.68, p > 0.05 (p = 0.19) for deoxy-Hb), though there is a trend that HB had lower correlation values than both MN and MN-WU.


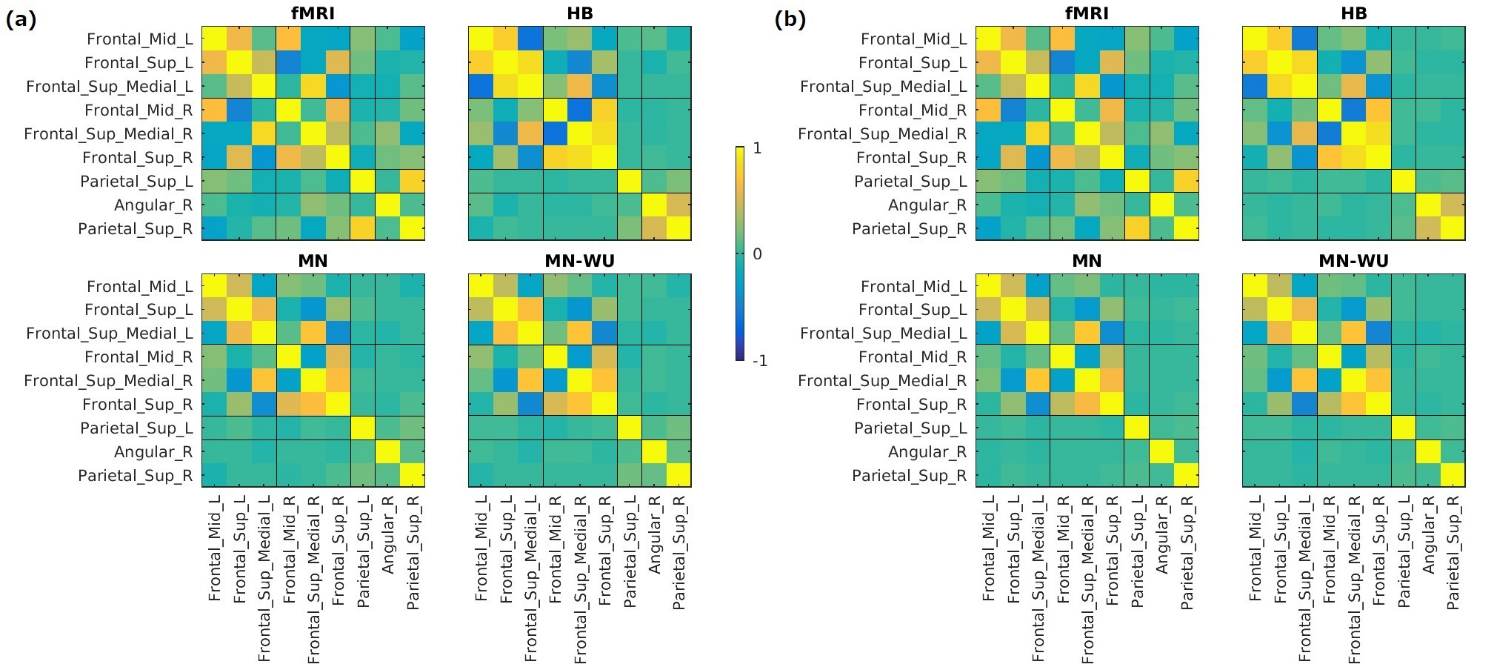


Figure S2. Mean maps for RSFCs of fMRI and DOT with HB, MN and MN-WU algorithms. Correlation matrices were averaged across subjects and runs, for (a) oxy-Hb and (b) deoxy-Hb in the case of DOT.


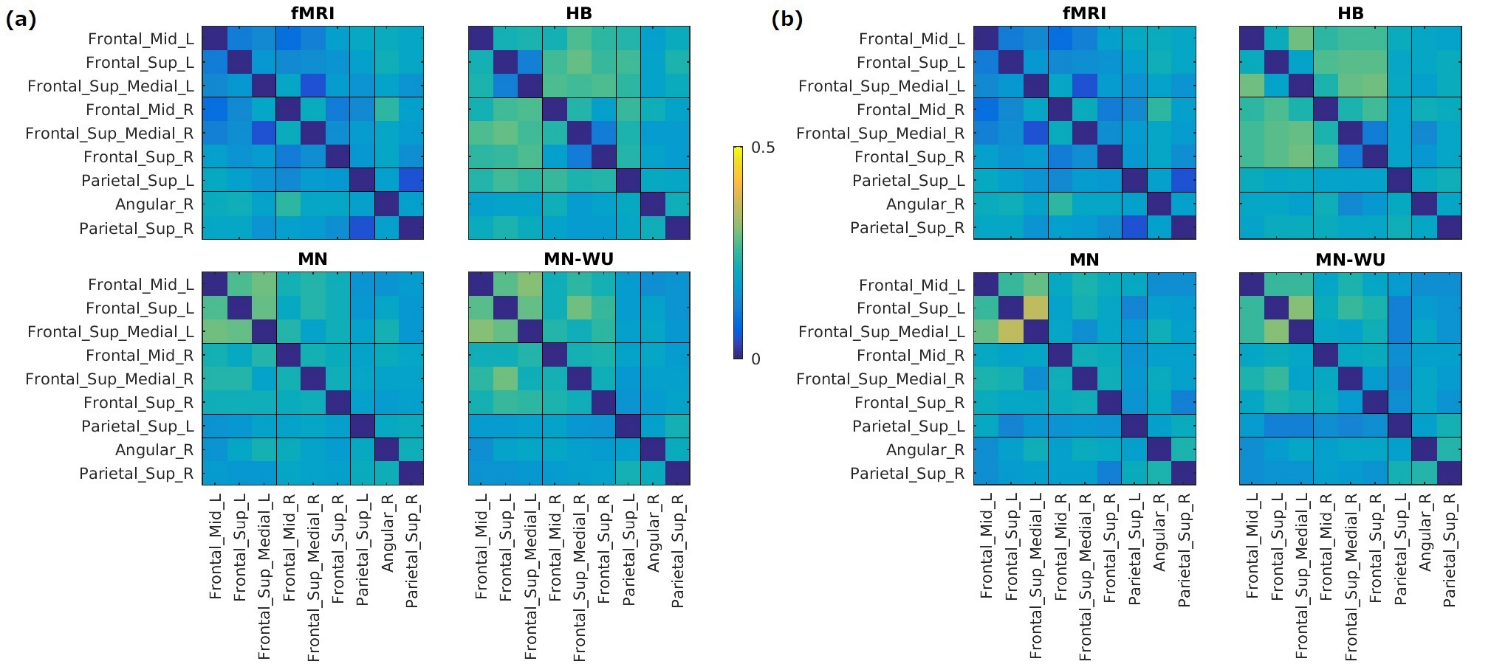


Figure S3. SD maps for RSFCs of fMRI and DOT with HB, MN and MN-WU algorithms. Correlation matrices were averaged across subjects and runs, for (a) oxy-Hb and (b) deoxy-Hb in the case of DOT.

Table S1. Similarity of correlation matrices between fMRI and DOT, in case of AAL. Values of correlation coefficients are presented as mean ± standard deviation (SD).

|  | | HB | MN | MN-WU |
| --- | --- | --- | --- | --- |
| oxy-Hb | run 1 | 0.49 ± 0.12 | 0.53 ± 0.11 | 0.49 ± 0.11 |
|  | run 2 | 0.45 ± 0.12 | 0.51 ± 0.12 | 0.51 ± 0.13 |
| deoxy-Hb | run 1 | 0.44 ± 0.12 | 0.50 ± 0.10 | 0.48 ± 0.11 |
|  | run 2 | 0.43 ± 0.15 | 0.47 ± 0.12 | 0.47 ± 0.13 |

**2 The AAL counterpart; inter-run reproducibility**

Mean and SD maps for RSFCs are compared between runs in Fig. S4 and S5, respectively. The inter-run reproducibility (i.e., Pearson’s correlation and inter-class correlation of correlation matrices between run 1 and 2) is summarized in Table S2.

As for Pearson’s correlation, one-way ANOVA revealed a significant difference among three DOT algorithms (F_(2,57)_ = 7.93, p < 0.001 (p = 0.0009) for oxy-Hb; F_(2,57)_ = 5.57, p < 0.01 (p = 0.0062) for deoxy-Hb). For oxy-Hb, the post-hoc Tukey’s HSD test revealed that HB had significantly higher correlation values then both MN and MN-WU did (p < 0.05), but the difference of correlation values between MN and MN-WU was not significant (p > 0.05). For deoxy-Hb, HB had significantly higher correlation values than MN (p < 0.05), but the difference of correlation values between HB and MN-WU and between MN and MN-WU was not significant (p > 0.05).

As for the ICC metrices, the following results were obtained. First, for ICC(C,1), one-way ANOVA revealed a significant difference among three DOT algorithms for oxy-Hb (F_(2,57)_ = 4.63, p = 0.0137), but not for deoxy-Hb (F_(2,57)_ = 2.24, p = 0.1159). For oxy-Hb, the post-hoc Tukey’s HSD test revealed that HB had significantly higher ICC(C,1) values than MN did (p < 0.05), but the ICC(C,1) value for MN-WU was not significantly different from those of both HB and MN.

Then, for ICC(C,k), one-way ANOVA revealed a significant difference among three DOT algorithms for oxy-Hb (F_(2,57)_ = 4.06, p = 0.0224), but not for deoxy-Hb (F_(2,57)_ = 1.23, p = 0.3002). For oxy-Hb, the post-hoc Tukey’s HSD test revealed that HB had significantly higher ICC(C,k) values than MN did (p < 0.05), but the ICC(C,k) value for MN-WU was not significantly different from those of HB and MN.


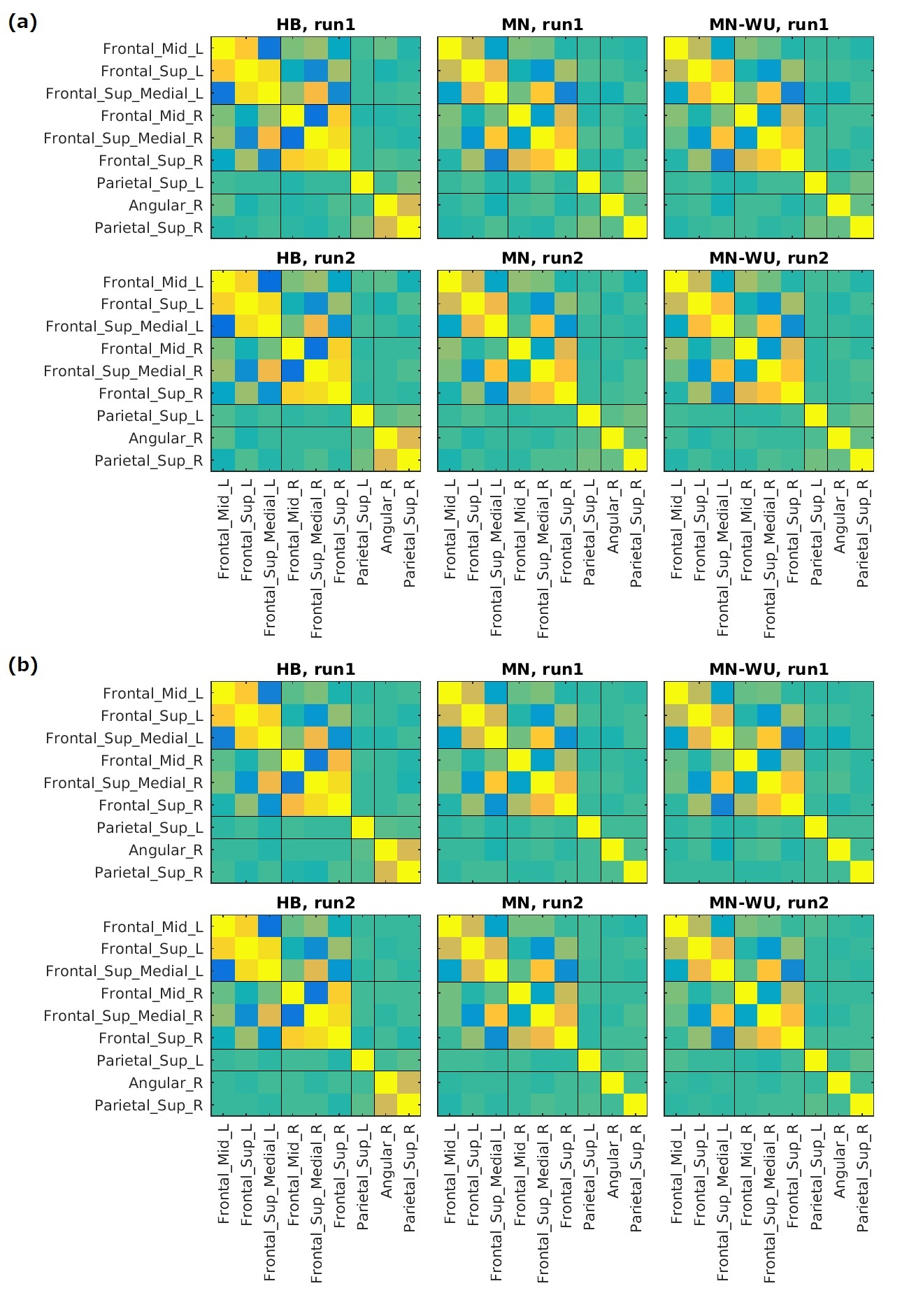


Figure S4. Mean maps for RSFCs are compared between run 1 and run 2. Correlation matrices are averaged across subjects for (a) oxy-Hb and (b) deoxy-Hb. The upper and lower rows correspond to run 1 and run 2, respectively.


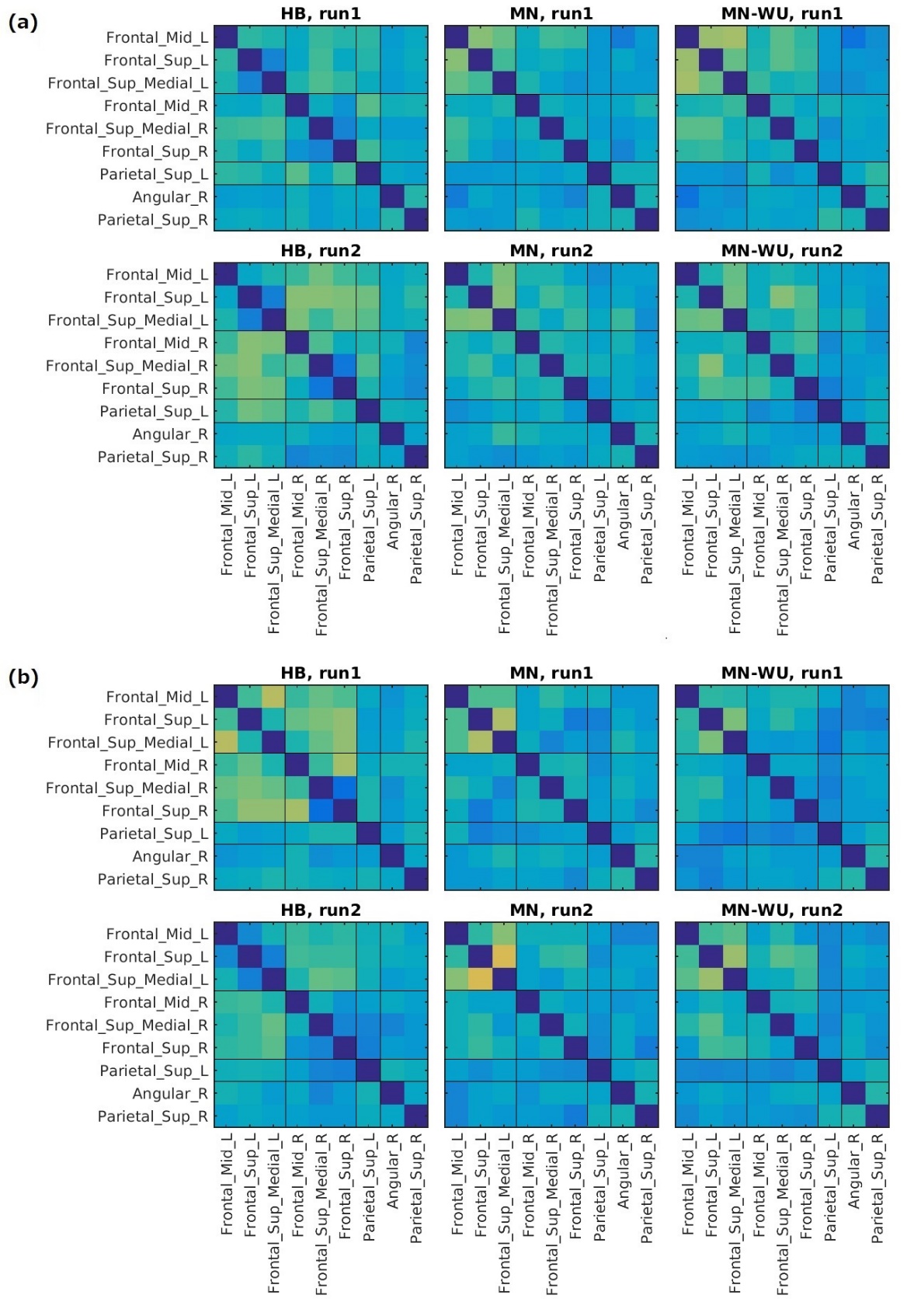


Figure S5. SD maps for RSFCs are compared between run 1 and run 2. Correlation matrices are averaged across subjects for (a) oxy-Hb and (b) deoxy-Hb. The upper and lower rows correspond to run 1 and run 2, respectively.

Table S2. Inter-run reproducibility. Values of Pearson’s correlation (r), ICC(C,1) and ICC(C,k) between runs are presented as mean ± standard deviation (SD).

|  | | HB | MN | MN-WU |
| --- | --- | --- | --- | --- |
| oxy-Hb | r | 0.88 ± 0.08 | 0.80 ± 0.08 | 0.82 ± 0.09 |
|  | ICC(C,1) | 0.87 ± 0.08 | 0.79 ± 0.08 | 0.81 ± 0.10 |
|  | ICC(C,k) | 0.93 ± 0.05 | 0.88 ± 0.05 | 0.89 ± 0.07 |
| deoxy-Hb | r | 0.85 ± 0.14 | 0.78 ± 0.10 | 0.82 ± 0.08 |
|  | ICC(C,1) | 0.84 ± 0.15 | 0.77 ± 0.10 | 0.81 ± 0.08 |
|  | ICC(C,k) | 0.90 ± 0.12 | 0.86 ± 0.07 | 0.89 ± 0.05 |

**3 Kendall’s W analysis**

As mentioned in the main text, timeseries were averaged across all sensitive voxels within each ROI in the calculation of RSFCs. This is based on the assumption that mean timeseries represent temporal activity of the corresponding ROI because each region of the Shen’s atlas includes a single functional area. To confirm this assumption, we conducted the following analysis.

First, for each ROI, we calculated Kendall’s coefficient of concordance *W* as follows. Suppose that there are in total *n* time points and *m* voxels in the ROI. For *j*-th voxel, activity values are rated in rank order from *1* to *n*, and let *r_ij_* be the rank for the *j*-th voxel at *i*-th time. Then the total rank given to time *i* is $R_{i}=\sum_{j=1}^{m} r_{ij}$ and the mean value of these total ranks is $\bar{R}=\frac{1}{n}\sum_{i=1}^{n} R_{i}$ . The sum of squared deviations, *S*, is defined as $S=\sum_{i=1}^{n} {(R_{i}-\bar{R})}^{2}$ , and then the Kendall’s *W* is defined as $W= \frac{12S}{m^{2}(n^{3}-n)}$ . If the test static *W* is 1, then the timecourses of all voxels in the corresponding ROI are the same. If *W* is 0, then there is no overall trend of agreement among the various voxels in the ROI. Under the null hypothesis that there is no concordance among all voxels in the corresponding ROI, we conducted chi-squared test for each ROI, where chi-squared is defined as $\text{χ}^{2}=m\left( n-1 \right)W$. If the null hypothesis is rejected for all ROIs, then it is appropriate to consider that the mean timeseries represent temporal activity of the corresponding ROI.

Next, we compared *W* averaged among all *available* ROIs between Shen’s atlas and AAL atlas with two-sample t-test. If Shen’s atlas has greater W value than AAL atlas does, then it is appropriate to use Shen’s atlas.

The results of Kendall’s W analysis are as follows.

For all subjects, the chi-squared test under the null hypothesis that there is no concordance among all voxels in the corresponding ROI revealed *p < 0.05* for all *available* ROIs. This is true irrespective of the DOT algorithms. Thus, the null hypothesis is rejected and therefore it is appropriate to consider that the mean timeseries represent temporal activity of the corresponding ROI.

Next, two-sample t-test revealed that the *W* calculated from ROI activities estimated with the HB DOT is significantly larger for Shen’s atlas than for AAL atlas for both oxy- and deoxy-Hb (p = 2.64×10^-23^ for oxy-Hb; p = 3.85×10^-24^ for deoxy-Hb). This is true for both MN (p = 1.52×10^-25^ for oxy-Hb; p = 9.50×10^-24^ for deoxy-Hb) and MN-WU (p = 6.48×10^-23^ for oxy-Hb; p = 3.48×10^-22^ for deoxy-Hb). Thus, regardless of the DOT algorithms, *W* for the Shen’s atlas is larger than that for the AAL atlas, supporting the validity of using the Shen’s atlas in the calculation of functional connectivity.

Table S3. Results of Kendall’s W analysis. Values of *W* are presented as mean±SD. The upper and lower rows in each cell correspond to oxy- and deoxy-Hb, respectively.

|  | | HB | MN | MN-WU |
| --- | --- | --- | --- | --- |
| Shen | oxy-Hb | 0.43±0.19 | 0.30±0.15 | 0.31±0.15 |
|  | deoxy-Hb | 0.42±0.19 | 0.27±0.13 | 0.27±0.13 |
| AAL | oxy-Hb | 0.27±0.09 | 0.17±0.06 | 0.19±0.07 |
|  | deoxy-Hb | 0.26±0.09 | 0.16±0.07 | 0.17±0.08 |

**4 Principal component analysis**

We conducted principal component analysis (PCA) to further confirm the validity of using voxel-averaged timeseries. For each region of the Shen’s atlas (and also AAL atlas), PCA was applied to timeseries of all the *sensitive* voxels in the ROI. We focused on the first principal component (PC1), and investigated (1) percent variance explained by PC1 and (2) similarity between PC1 and voxel-averaged timeseries. If percent variance explained by PC1 is large, most of voxels in each ROI may be considered to behave in a similar fashion. In addition, if similarity between PC1 and voxel-averaged timeseries is high, it would be appropriate to consider that voxel-averaged timeseries represent temporal activity of the corresponding ROI. As a similarity measure, we used the correlation coefficient between PC1 and voxel-averaged timeseries.

Table S4 summarizes the results for the Shen’s atlas. First, regardless of DOT algorithms, mean percent variance explained by PC1 is more than about 70 %, indicating that the PC1 can explain the majority of the component contained in hemodynamics of all the *sensitive* voxels in the ROI. Second, regardless of DOT algorithms, mean correlation coefficient is more than 0.90, indicating that the voxel-averaged timeseries is similar to the PC1 timeseries. These results confirm the validity of using voxel-averaged timeseries as a representative.

On the other hand, the results for the AAL atlas are shown in Table S5. Regardless of DOT algorithms, the percent variance in the Shen’s atlas case is significantly greater than that in the AAL atlas case, for both oxy- and deoxy-Hb (two-sample t-test, p < 0.05). In addition, regardless of DOT algorithms, the correlation coefficient in the Shen’s atlas case is significantly higher than that in the AAL atlas case, for both oxy- and deoxy-Hb (two-sample t-test, p < 0.05). These results confirm the validity of using Shen’s atlas.

Table S4. Results of the analysis with PCA for Shen’s atlas. The upper row shows the percent variance explained by PC1. The lower row shows the correlation coefficient between PC1 and voxel-averaged timeseries. All data are presented as mean ± SD over all available ROIs, runs and subjects.

|  | | HB | MN | MN-WU |
| --- | --- | --- | --- | --- |
| Percent variance | oxy-Hb | 91.3±7.19 | 72.2±13.7 | 73.9±13.5 |
|  | deoxy-Hb | 90.3±7.30 | 69.2±14.2 | 69.7±14.1 |
| Correlation coefficient | oxy-Hb | 0.99±0.03 | 0.95±0.17 | 0.95±0.15 |
|  | deoxy-Hb | 0.99±0.03 | 0.92±0.24 | 0.92±0.20 |

Table S5. Results of the analysis with PCA for AAL atlas. The upper row shows the percent variance explained by PC1. The lower row shows the correlation coefficient between PC1 and voxel-averaged timeseries. All data are presented as mean ± SD over all available ROIs, runs and subjects.

|  | | HB | MN | MN-WU |
| --- | --- | --- | --- | --- |
| Percent variance | oxy-Hb | 82.9±11.0 | 53.3±13.9 | 57.1±14.2 |
|  | deoxy-Hb | 80.7±11.1 | 50.2±13.8 | 53.0±13.7 |
| Correlation coefficient | oxy-Hb | 0.98±0.05 | 0.85±0.27 | 0.85±0.27 |
|  | deoxy-Hb | 0.97±0.09 | 0.76±0.33 | 0.74±0.33 |
